# Wood’s Medical and Surgical Monographs

**Published:** 1889-05

**Authors:** 


**Wood's Medical and Surgical Monographs.** Vol. I., No. 2, February, 1889. Vol. I., No. 3, March, 1889. Vol. II., No. 1, April, 1889. Published monthly. Price, $10 a year; single copies, $1.00. New York: William Wood & Company, 56 and 58 Lafayette place.

We have already noticed the first article in the February issue of these valuable monographs, viz.: "Gonorrheal Infection in Women," by William Japp Sinclair, M. A., M. D., promising to touch upon the remaining titles in this number at a future time.

The second article on "Giddiness," by Thomas Grainger Stewart, M. D., consists of three lectures upon this most important symptom of intra-cranial disturbance. In Lecture I., he considers definitions, causes, varieties, and relationship to diseases of the semi-circular canals. In his description of peripheral giddiness, which he asserts is usually due to *contradictoriness of sensory impression*, he refers to the giddiness that most persons experience in visiting the Leaning Tower of Pisa, and which he himself experienced when ascending or descending its stairs.

In Lecture II. is given an account of the discovery and pathology of Menière's Disease—Labyrinthine Vertigo. This is illustrated with some cases of rare interest. The final lecture treats of giddiness as a symptom in cases of disease of the Pons Varolii. These lectures possess rare interest to the otologist, neurologist, and diagnostician.

The closing article of this number, "A Critical Study of the Chemical Value of Albuminuria in Bright's Disease," by Dr. Pierre Jaenton, will be found of great interest in connection with the recent publications of Stewart, Loomis, and others on kindred subjects. The interest of the profession in diseases of the kidney is readily determined to be relatively great, from the mass of literature that is accumulating thereon. This author is strong in his description of the methods of determining albumin in the urine.

Vol. I., No. 3, March, 1889, "Neurasthenia and Its Treatment," by Dr. H. Von Ziemssen, is a most interesting consideration of an American disease by a German author. It is needless to say that full credit is given to Beard, who was the first to describe the group of symptoms arising from overworking of the nervous system under the disease-term neurasthenia. The author's description of his typical cases of worked-out American neurasthenics is both graphic and amusing.

"Antipyresis, and Antipyretic Methods of Treatment" are next considered by the same author. Most Americans have already become familiar with Dr. Von Ziemssen's views, of twelve or fifteen years ago, on fever, as published in the Cyclopedia which has made his name famous in this country. In the present paper he brings himself forward to the present standpoint of bacteriological investigation, though still rigidly adhering to hydrotherapy in typhoid—a point of view that has never taken firm hold on this side of the Atlantic.

"The Tongue as an Indication of Disease," receives masterly handling in an introduction and three lectures, by W. Howship Dickinson, M. D., etc. This author has already made himself familiar to Americans through his "Treatise on Albuminuria," published in Wood's Library in 1881. This latest appearance only serves to strengthen his large audience in the former good opinion that it formed of him. Appropriate illustrations on wood, and in color, serve to elucidate the text, and it is presumed that every progressive physician will possess himself of this attractive number.

"The Treatment of Cystic Goitre" is a subject of much interest to surgeons, who will all be interested to know what Mr. Thomas M. Hovell has to say thereon. This, too, is illustrated by cuts of interesting cases, that show the results of treatment. The author considers the various methods of treatment advocated by different surgeons, and adopts Sir Morell Mackenzie's plan of injecting the cavity, after withdrawing its contents, with a solution of the perchloride of iron as the safest and best yet proposed.

Under the head of New Remedies, Dr. C. Cauquil considers the principal medicinal agents introduced into the materia medica from 1878 to 1888. This, together with the new edition of Ringer, equips the physician with the latest literature of this subject extant.

A title-page and table of contents for the three numbers serve to make Volume I. ready for the binder.

The first number of Volume II, for April, 1889, is one of unusual interest. The essay on "Diabetes and its Connection with Heart Disease," by Jaques Meyer, M. D., occupies the first twenty-nine pages of the book, and will receive examination on account of the paucity of literature on this subject.

The remainder of the book, 272 pages, is devoted to the consideration of "Blenorrhea of the Sexual Organs and its Complications," by Dr. Ernest Finger, Docent of the University of Vienna. In the introduction are considered the history and etiology of the disease. This is a most interesting portion of the work, and deserves careful study. His description of the gonococcus of Neisser is simple, clear, and convincing. Finger commits himself unhesitatingly to the opinion that blenorrhagic urethritis can only develop by inoculation with gonococci.

Whoever studies this treatise carefully, will surely obtain some new knowledge on this most distracting and unsatisfactory subject. He will here find why this disease has proven so intractable in its management, and the way to overcome many difficulties that lie in the path of cure; he will here obtain new inspiration for the combat with this dread malady—one that is all too common, and that is doing more damage to both sexes, directly and indirectly, than any other disease of the sexual system.

It has ceased to be a slight responsibility to undertake the cure of a clap, and it is high time both physicians and laity were so taught. Patients, male and female, must be informed of the serious nature of their ailment when once infected, and be placed under the most rigid sexual quarantine. In short, the methods of management that have heretofore prevailed to a considerable extent must be revolutionized and placed upon the highest scientific plane. This work of Finger and that of Sinclair should be studied together by every physician who would do himself and his clients justice in this field.

